# Management of Abdominal Aortic Aneurysm With Concomitant Acute Cholecystitis in the Era of Endovascular Surgery

**DOI:** 10.7759/cureus.37433

**Published:** 2023-04-11

**Authors:** Bradley Trinidad, Sirin Falconi, Nathan Kragh, Muhammad Nazim

**Affiliations:** 1 Department of Surgery, Texas Tech University Health Sciences Center, Amarillo, USA; 2 Department of Surgery, Northwest Texas Hospital, Amarillo, USA; 3 School of Medicine, Texas Tech University Health Sciences Center, Amarillo, USA

**Keywords:** acute calculus cholecystitis, laparoscpic surgery, concomitant vascular and general surgery, aortobiiliac graft, endovascular surgical repair, endovascular abdominal aortic aneurysm repair

## Abstract

The incidence of symptomatic acute cholecystitis with large (greater than 5.5 cm) abdominal aortic aneurysm is an uncommon occurrence. Guidelines on concomitant repair in this setting remain elusive, particularly in the era of endovascular repair. We present a case of acute cholecystitis in a 79-year-old female presenting to a local rural emergency room with abdominal pain and known abdominal aortic aneurysm (AAA). Abdominal computed tomography (CT) revealed a 5.5 cm infrarenal abdominal aortic aneurysm, significantly greater in size compared to previous imaging, as well as a distended gallbladder with mild wall thickening and cholelithiasis concerning for acute cholecystitis. The two conditions were found to be unrelated to each other, but concerns were raised on appropriate timing of care. Following diagnosis, the patient underwent concomitant treatment of acute cholecystitis and large abdominal aortic aneurysm with laparoscopic and endovascular techniques, respectively. In this report, we take the opportunity to discuss the treatment of patients with AAA and concomitant symptomatic acute cholecystitis.

## Introduction

An abdominal aortic aneurysm (AAA) is a pathologic dilation of the abdominal aorta with a diameter greater than 3 cm [1-4]. AAAs are commonly asymptomatic; therefore, screening with ultrasound is recommended for individuals aged 65 years and older or with risk factors [1]. The purpose of management is to repair the aneurysm before rupture [2]. Elective repair is indicated if the AAA is greater than 5.5 cm in males and 5 cm in females [1-4]. The Society for Vascular Surgery practice guidelines recommend endovascular repair as the preferred repair over open repair because of its minimally invasive therapy [2,3]. Acute cholecystitis is caused by inflammation of the gallbladder due to cystic duct obstruction [5]. The prevalence of gallbladder disease is nearly 20 million individuals in the United States, with more than 200,000 people diagnosed with acute cholecystitis each year; of those, 10 to 15% have cholelithiasis and 80% are asymptomatic [5]. Ultrasound is the imaging modality of choice, and the majority are treated with laparoscopic cholecystectomy [5].

The incidence of concurrent symptomatic acute cholecystitis and large (greater than 5.5 cm) abdominal aortic aneurysm is uncommon. Guidelines on concomitant repair in this setting remain elusive, particularly in the era of endovascular repair. Herein is described a case of concomitant treatment of acute cholecystitis and abdominal aortic aneurysm using endovascular aortic aneurysm repair in a 79-year-old female presenting with vague abdominal pain.

## Case presentation

**Figure 1:**
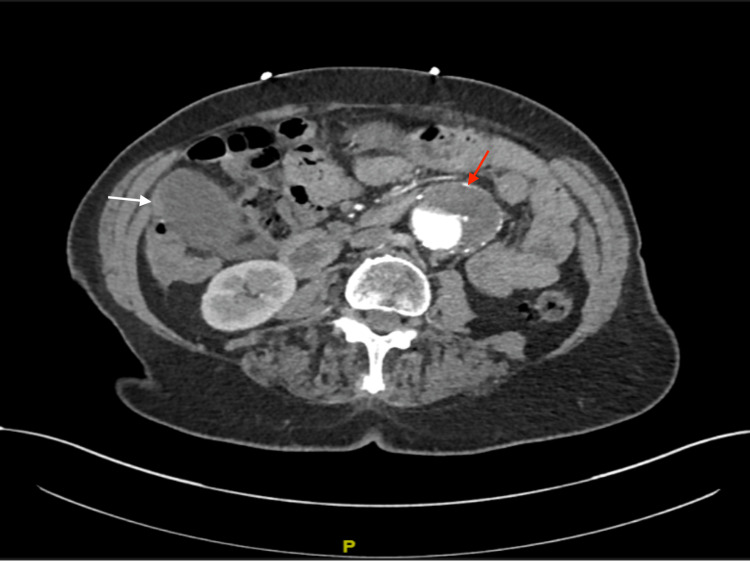
CTA abdomen and pelvis Computer tomography angiography (CTA) abdomen and pelvis showing concomitant 5.5 cm infrarenal abdominal aortic aneurysm and distended gallbladder with wall thickening concerning cholecystitis.

A 79-year-old female smoker with hypertension, gastroesophageal reflux disease (GERD), and known AAA presented to the local rural emergency department with worsening right upper quadrant and mid-epigastric pain with associated nausea and vomiting. She was evaluated with laboratory and radiological studies. In Figure 1, CT chest/abdomen/pelvis with intravenous contrast showed findings of a distended gallbladder with gallbladder wall thickening and gallstones concerning for acute cholecystitis (white arrow), as well as a 5.5 cm infrarenal abdominal aortic aneurysm (red arrow). The aneurysm had measured 4.9 cm six months prior. There was question of a potential symptomatic aneurysm.

The patient was transferred to our institution for vascular surgery evaluation. General surgery referral was also obtained. Gallbladder ultrasound (US) and hepatobiliary iminodiacetic acid (HIDA) scan were performed upon arrival, confirming the previous results. Laboratory data revealed mildly elevated liver enzymes and a normal ALP and total bilirubin. WBC was slightly elevated at 10.8 x 10^3^/mcL. Troponins were negative. Vitals signs were within normal limits. Physical exam showed tenderness to palpation in the right upper quadrant with “palpable gallbladder.” The aneurysm was not tender on four-point palpation. Per protocol, the patient was evaluated by cardiology with Lexiscan nuclear stress test and echocardiogram for risk stratification and was cleared for surgery. Blood cultures were taken on admission and were negative. Leukocytosis resolved on intravenous (IV) antibiotics. She remained afebrile. After three days of close observation and monitoring with continued negative blood cultures, she was taken to the hybrid operating suite for a laparoscopic cholecystectomy with lysis of adhesion followed by endovascular aortic aneurysm repair. This approach was used to limit the possibility of complications of either of the two untreated conditions in the case of separate treatment. The gallbladder was removed with a dome down approach. Intraoperatively, findings consisted of an extremely thickened, distended gallbladder with gangrenous appearance, along with extensive omental adhesions. Once complete, and while the patient was still under general anesthesia, percutaneous access to the bilateral common femoral arteries under ultrasound guidance was performed with subsequent endovascular repair of the infrarenal aorta by the deployment of an aortobiiliac endograft (Terumo, United Kingdom). The procedures were completed without complication. She remained on IV antibiotics throughout the perioperative period and was discharged on two weeks of oral antibiotic therapy on postoperative day seven. Upon follow up one month later, she remained well without complication. Repeat CT scan revealed no evidence of endoleak, infection, or intra-abdominal abscess.

## Discussion

The incidence of acute cholecystitis complicating standard abdominal aortic aneurysm (AAA) repair was described previously, with reports suggesting up to 18 percent [6]. This led to a more aggressive approach to treating asymptomatic cholelithiasis in patients with large abdominal aortic aneurysms requiring repair [7]. As endovascular repair of aneurysms became standard, combining repair with laparoscopic cholecystectomy for cholelithiasis was thought to be a safe and effective option [8,9]. However, more recent literature suggest endovascular repair of AAA does not appear to predispose patients to the development of symptomatic cholecystitis during the perioperative period in those with cholelithiasis, not necessitating cholecystectomy prior or concomitantly [10]. Less described, however, is the treatment of patients who present with acute cholecystitis and large abdominal aortic aneurysm greater than 5.5 cm, meeting requirements for repair, particularly in the era of endovascular aortic aneurysm repair. 

To date, only one other case report has described combined operative approach to large abdominal aortic aneurysm and acute cholecystitis. In that case, a patient with symptomatic cholecystitis and a 6.2 cm infrarenal abdominal aortic aneurysm was treated successfully with endovascular aortic aneurysm repair using an Endurant (Medtronic, Santa Rosa, CA) prosthesis, followed by laparoscopic cholecystectomy once clotting time normalized [11]. This case differs from that presented above, in which gallbladder removal was performed first, prior to endovascular aneurysm repair (EVAR). We felt this to be the safest approach so as to reduce potential kinking or malpositioning of the newly placed graft during CO_2_ insufflation, as well as the ability to abort the planned EVAR in setting of accidental gross spillage. There remains no data on the appropriate timing of the interventions. Other authors have suggested low risk of graft kinking or migration by pneumoperitoneum secondary to suprarenal fixation and increased radial force of newer generation grafts [11].

The biggest concern in these cases remains infection risk in the setting of active acute cholecystitis. Aortic graft infection is a serious complication associated with significant morbidity and mortality [12,13]. It is hypothesized that the contamination potential in the setting of endovascular repair will be low given that no gallbladder contents come into contact with the prosthesis at any time, nor is there direct access between the two spaces. However, previous studies found that there is an 18% incidence of perioperative biliary leakage in patients who do not undergo AAA repair with concomitant cholecystectomy [6]. Therefore, risk of graft infection should be weighed against the early and late complications of leaving a diseased gallbladder in place [6]. Nonetheless, long-term empiric antibiotic chemoprophylaxis throughout the perioperative period was employed in both the described cases above, along with the establishment of multiple days of negative blood cultures. Currently, there is no literature that exists to guide length of treatment, but a two-week postoperative course was felt sufficient, if not overkill, in the case above.

Finally, the question of whether EVAR can be deferred until after laparoscopic cholecystectomy has been raised. This remains controversial as there is a theoretically increased risk for rupture postoperatively secondary to theorized increase in collagenase activity [14,15]. In our case, after weighing the risks and benefits, it was determined safest to proceed concomitantly due to the presence of a large AAA and findings significant for symptomatic acute cholecystitis. However, it should be noted that this theory has been described predominantly with laparotomy and has not been defined as clearly in laparoscopic techniques.

## Conclusions

An abdominal aortic aneurysm is a disease process with significant morbidity and mortality if not treated appropriately. Acute cholecystitis, although fairly common, can become an alarming complication in the treatment of AAA. When these two disease processes occur concurrently, prompt treatment is key and should not be delayed due to the high risk of complications. Concomitant treatment of acute cholecystitis and abdominal aortic aneurysm with laparoscopic and endovascular techniques, respectively, appears to be a safe and prudent approach in cases where gallbladder disease is significant and the risk of further aggravating a concomitant AAA is high, though further study is needed to establish standard of care. Finally, a case-by-case approach should be employed in patients presenting with AAA and concomitant gallbladder disease with evaluation of the risks and benefits of pursuing a concomitant approach.
